# Four Novel Botourmiaviruses Co-Infecting an Isolate of the Rice Blast Fungus *Magnaporthe oryzae*

**DOI:** 10.3390/v12121383

**Published:** 2020-12-03

**Authors:** Yang Liu, Liyan Zhang, Ahmed Esmael, Jie Duan, Xuefeng Bian, Jichun Jia, Jiatao Xie, Jiasen Cheng, Yanping Fu, Daohong Jiang, Yang Lin

**Affiliations:** 1 State Key Laboratory of Agricultural Microbiology, Huazhong Agricultural University, Wuhan 430070, China; liu813801@gmail.com (Y.L.); ahmed.esmael@fsc.bu.edu.eg (A.E.); jiajichun@webmail.hzau.edu.cn (J.J.); jiataoxie@mail.hzau.edu.cn (J.X.); daohongjiang@mail.hzau.edu.cn (D.J.); 2 Institute of Biotechnology, Heilongjiang Academy of Agricultural Sciences, Harbin 150001, China; zhangliyanyong@126.com; 3 The Provincial Key Lab of Plant Pathology of Hubei Province, College of Plant Science and Technology, Huazhong Agricultural University, Wuhan 430070, China; duanjie@webmail.hzau.edu.cn (J.D.); xuefengbian@webmail.hzau.edu.cn (X.B.); jiasencheng@mail.hzau.edu.cn (J.C.); yanpingfu@mail.hzau.edu.cn (Y.F.); 4 Botany and Microbiology Department, Faculty of Science, Benha University, Qalubiya Governorate, Benha 13511, Egypt

**Keywords:** +ssRNA, botourmiavirus, MOBV2, MOBV5, MOBV6, MOBV7, *Magnaporthe oryzae*

## Abstract

Via virome sequencing, six viruses were detected from *Magnaporthe oryzae* strains YC81-2, including one virus in the family *Tombusviridae*, one virus in the family *Narnaviridae* and four viruses in the family *Botourmiaviridae*. Since the RNA-dependent RNA polymerase (RdRp) of one botourmiavirus show the highest identity (79%) with Magnaporthe oryzae ourmia-like virus 1 (MOLV1), the virus that was grouped into the genus *Magoulivirus* was designated as Magnaporthe oryzae botourmiavirus 2 (MOBV2). The three other novel botourmiaviruses were selected for further study. The complete nucleotide sequences of the three botourmiaviruses were determined. Sequence analysis showed that virus 1, virus 2, and virus 3 were 2598, 2385, and 2326 nts in length, respectively. The variable 3′ untranslated region (3′-UTR) and 5′-UTR of each virus could be folded into a stable stem-loop secondary structure. Each virus consisted of a unique ORF encoding a putative RdRp. The putative proteins with a conserved GDD motif of RdRp showed the highest sequence similarity to RdRps of viruses in the family *Botourmiaviridae*. Phylogenetic analysis demonstrated that these viruses were three distinct novel botourmiaviruses, clustered into the *Botourmiaviridae* family but not belonging to any known genera of this family. Thus, virus 1, virus 2, and virus 3 were designated as Magnaporthe oryzae botourmiavirus 5, 6, and 7 (MOBV5, MOBV6, and MOBV7), respectively. Our results suggest that four distinct botourmiaviruses, MOBV2, MOBV5, MOBV6, and MOBV7, co-infect a single strain of *Magnaporthe oryzae*, and MOBV5, MOBV6, and MOBV7 are members of three unclassified genera in the family *Botourmiaviridae*.

## 1. Introduction

Mycoviruses, viruses of fungi, are ubiquitous and diverse in all major phyla of fungi [1]. They can cause a variety of different consequences after infecting their hosts. Most mycoviruses usually remain latent in their host without causing any symptoms [1], while a few mycoviruses cause a series of debilitating symptoms, including irregular growth, abnormal pigmentation, altered sexual reproduction, attenuation of virulence (hypovirulence), and so on [1]. A few mycoviruses even confer increased virulence (hypervirulence) to their hosts [2,3].

The genomes of most mycoviruses are double-stranded RNA (dsRNA) or linear positive-sense single-stranded RNA (+) ssRNA [4]. Only a few mycovirus genomes have been reported to consist of linear negative-sense single-stranded RNA (–) ssRNA [5,6], or circular single-stranded DNA (ssDNA) [7,8,9,10]. As novel unclassified mycoviruses are constantly being discovered, the taxonomy of mycoviruses is frequently being updated. So far, known linear (+) ssRNA mycoviruses have been classified into six families: *Alphaflexiviridae*, *Barnaviridae*, *Gammaflexiviridae*, *Deltaflexiviridae*, *Narnaviridae*, and *Botourmiaviridae* [5,11]. Among them, *Botourmiaviridae*, a new family established by the International Committee on the Taxonomy of Viruses (ICTV) [11], is divided into four genera: one plant virus genus, *Ourmiavirus,* including plant ourmiaviruses, and three mycovirus genera, *Scleroulivirus*, *Botoulivirus*, and *Magoulivirus,* that include botourmiaviruses (previously named ourmia-like mycoviruses). However, several botourmiaviruses do not belong to the above three mycovirus genera, and have been clustered into an undefined clade.

The first mycovirus in *Magnaporthe oryzae*, the pathogen of rice blast, was discovered in 1971 [12]. Since then, mycoviruses in six families have been found to be associated with *M. oryzae*. Of these mycoviruses, dsRNA viruses are clustered into three families: Magnaporthe oryzae virus 1, 2, and 3 (MoV1, MoV2, and MoV3) are classified into the family *Totiviridae* [13,14,15]. Magnaporthe oryzae chrysovirus 1A, B, C, and D (MoCV1-A, MoCV1-B, MoCV1-C, MoCV1-D) are grouped into the family *Chrysoviridae* [14,16,17,18]. Magnaporthe oryzae partitivirus 1 [19], 2 [20], and 3 belong to the family *Partitiviridae*. In addition, several (+) ssRNA mycoviruses have also been found to be associated with *M. oryzae*. Among them, Magnaporthe oryzae virus A is closely related to plant viruses of the family *Tombusviridae*. Magnaporthe oryzae ourmia-like virus 1 (MOLV1) and Magnaporthe oryzae ourmia-like virus 4 (MOLV4) are in the family *Botourmiaviridae* [21,22,23]. Another three botourmiaviruses, Pyricularia oryzae ourmia-like viruses 1 to 3, have been identified from the *P. oryzae* isolate infecting wheat [24]. Recently, the first narnavirus infecting *M. oryzae,* in the family *Narnaviridae*, Magnaporthe oryzae narnavirus virus 1 (MoNV1), was identified [25].

To the best of our knowledge, only four dsRNA mycoviruses in two families, *Chrysoviridae* and *Partitiviridae,* have been reported to impact the the biological characteristics of their fungal host, *M. oryzae*. Among them, three chrysoviruses, MoCV1-A, MoCV1-B, and MoCV1-D, confer hypovirulence to *M. oryzae*. The infection of these hypovirulent viruses diminishes the pathogenicity of their fungal hosts by changing the biological characteristics of *M. oryzae,* such as by impairing hyphal growth, causing abnormal colony morphology, and reducing pigmentation [16]. In addition, Magnaporthe oryzae partitivirus 2 (MOPV2) has also been identified to be associated with the conidiation and pathogenicity of *M. oryzae* [20]. After infection with MOPV2, *M. oryzae* sporulation and virulence to barley substantially decreased. However, the effects of the (+) ssRNA mycovirses from three families, *Tombusviridae*, *Narnaviridae*, and *Botourmiaviridae,* on *M. oryzae* have not been previously mentioned.

A single fungal strain can generally be infected by multiple mycoviruses. This kind of co-infection can be facilitated by chemical compound- or virus-mediated suppression of heterogenic recognition and RNA silencing [26,27,28,29]. In addition to being used to study the diversity of mycoviruses and the virus–fungus interaction, fungi that are co-infected by multiple mycoviruses are also ideal materials for studying the interaction between viruses [30]. So far, several virus–virus interactions have been recognized, such as synergistic interactions [31,32], mutualistic interactions [33,34,35,36], antagonistic interactions [27], and induced genome rearrangement [37,38,39]. There is also some evidence that the co-infections of mycoviruses with distantly related partners are more stable than that of related viruses [40,41].

In this study, to expand the knowledge of mycoviral diversity in *M. oryzae*, we isolated and characterized four novel (+) ssRNA botourmiaviruses, designated as Magnaporthe oryzae botourmiavirus 2 (MOBV2), MOBV5, MOBV6, and MOBV7, from a single *M. oryzae* strain, YC81-2, that was isolated from a field in China. Sequence evaluations and phylogenetic analysis of their RdRps showed that MOBV2 belongs to the genus *Magoulivirus*, while MOBV5, MOBV6, and MOBV7 are clustered into three undefined clades.

## 2. Materials and Methods

### 2.1. Fungal Isolates and Growth Conditions

Diseased rice leaf samples with the typical symptoms of rice blast were collected from a rice field in Yichang, Hubei province, P. R. China. Single-spore (asexual spore) isolation was performed from lesions of the diseased rice leaf samples. Then, a single-spore isolate, YC81-2, was selected for further study. A virus-free *M. oryzae* strain, P131, was used as a control [42]. The colony morphology of the strains YC81-2 and P131 on Potato Dextrose Agar (PDA) was observed at 7 and 9 days after inoculation (dai). Both strains were also incubated at 28 °C on PDA covered with cellophane for 5 days, and hyphal morphology was observed by a light microscope. The colony diameters of the two strains were recorded at 3, 5, 7, and 9 dai to calculate the growth rate of each strain. To harvest mycelia for nucleic acid extraction, strain YC81-2 was incubated on PDA covered with cellophane membrane at 28 °C for 7 days. All strains were stored on PDA slants at 4 °C.

### 2.2. RNA Extraction and Purification

For RNA extraction, a fresh mycelial sample of strain YC81-2 was ground into fine powder in liquid nitrogen. To detect RNA viruses in strain YC81-2, dsRNA extraction was performed as previously described [43]. The dsRNA was analyzed by electrophoresis using 1.5% agarose gel. All dsRNA samples were treated with both DNase I and S1 nuclease before electrophoresis. Total RNA was extracted using Trizol reagent (Invitrogen, Carlsbad, CA, USA), according to the manufacturer’s protocol. RNase-free DNase was used to remove DNA contaminations. Total RNA was dissolved in sterilized diethylpyrocarbonate (DEPC) water and was stored at −80 °C. The concentration of the purified RNA was measured using a Thermo Scientific™ NanoDrop 2000 Spectrophotometer (Wilmington, DE, USA). The quality of total RNA was detected by agarose gel electrophoresis.

### 2.3. cDNAs Cloning and Sequencing

Virome sequencing was carried out on the Illumina HiSeq2000/2500 platform by GENEWIZ Technology Services (Suzhou, China). In brief, one microgram of total RNA was used to obtain ribosomal-depleted RNA and construct a cDNA library using a VAHTSTM Total RNA-seq (H/M/R) Library Prep Kit for Illumina (Vazyme Biotech Co., Nanjing, China). After sequencing, adapter-polluted, contaminated, paired-end reads shorter than 100 bp, low quality, high content of unknown base (N) reads, and the RNA and DNA sequences of *M. oryzae* were filtered out from the raw data. Then, de novo assembly was performed with CLC Genomics Workbench (version: 6.0.4). Primary UniGenes were spliced to generate final UniGenes using CAP3 EST. The final UniGenes were then subjected to BLAST, using BLASTx to search for homology with viral sequences against non-redundant (NR). The contigs corresponding to the same viruses were assembled to generate viral fragments by DNAMAN.

The ligase-mediated terminal amplification was performed to determine the terminal sequences of the viruses [44]. The adapter pC3-T7 loop (5′-p-GGATCCCGGGAATTCGGTAATACGACTCACTATATTTTTATAGTGAGTCGTATTA-OH-3′) was ligated to the 3′ and 5′ ends by T_4_ RNA ligase (Axygen, Wuhan, China). The PC3-T7 loop-ligated ssRNA was purified, denatured in dimethyl sulfoxide (Sigma-Aldrich, Shanghai, China), and then reversely transcribed into cDNA. The terminal sequences of the viral cDNA were amplified with the primer PC2 (5′-p-CCGAATTCCCGGGATCC-3′), a complementary primer to the PC3-T7 loop, and sequence-specific primer F1 or R1, corresponding to the 5′- or 3′-terminal sequences of all viral fragments. Then, using the first round PCR products as templates, primer pairs PC2/F2 and PC2/R2 were applied to conduct Nested PCR amplification of the 5′- and 3′-ends of the viruses, respectively. After purification with a gel extraction kit (Axygen, Wuhan, China), the purified PCR amplicons were cloned into the pMD18-T vector (TaKaRa, Dalian, China) and sequenced. The experiment was repeated three times independently. Primers used in this study are listed in Table S1.

### 2.4. Sequence Analysis

The ORFs of the viruses were predicted based on standard genetic code, and their homologous amino acid sequences were searched in the NCBI database, using ORF finder and the BLASTp programs, respectively. Sequence alignment was performed using the CLUSTALX 1.8 program [45]. Annotation of the conserved sequences was carried out using GeneDoc [46]. The percent identities matrix among the RdRp proteins of the botourmiaviruses was generated as Gilbert et al. described [47]. Percent identities were evaluated via multiple sequence alignment using Clustal Omega [48]. Then, the matrix was converted to heat map plots using a custom R script. A phylogenetic tree was constructed using MEGA software version 6.0 by the maximum likelihood (ML) method, with 1000 bootstrap replicates [49]. The secondary structures of the viral genome termini at the 5′ and 3′ ends were predicted via the RNAfold Webserver [50].

## 3. Results

### 3.1. Characteristics of Strain YC81-2

**Figure 1:**
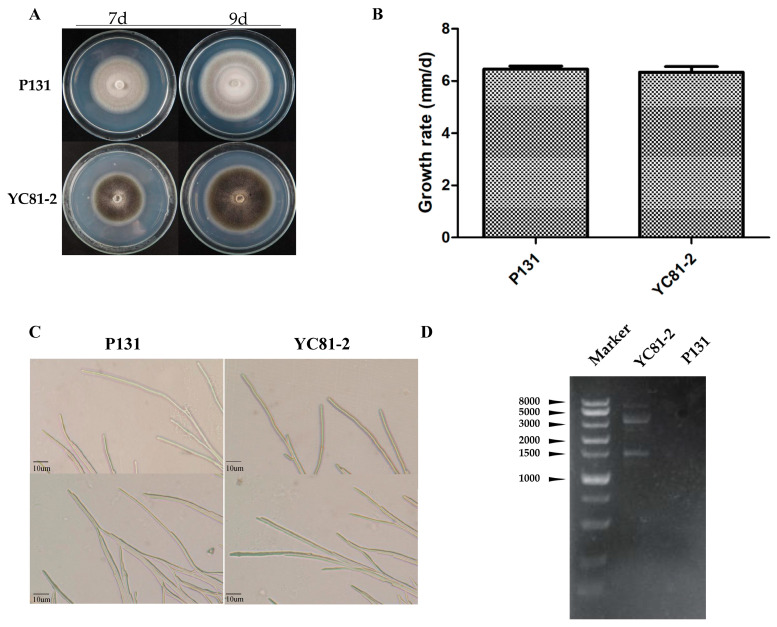
Morphological characteristics and dsRNA content of strains YC81-2 and P131. (**A**) Colony morphology of strain YC81-2 and P131 on PDA was observed at 7 and 9 days after inoculation (dai). (**B**) The growth rate was calculated by recording the colony diameters of the two strains at 3, 5, 7 and 9 dai, which showed that there was no significant difference between these two strains. Three independent replicates were performed. The values are means ± S.E. NS indicates no significance (*p* < 0.01, one-way ANOVA). (**C**) Hyphal morphology of strains YC81-2 and P131. Both strains were incubated at 28 °C on PDA covered with cellophane for 5 days, and the hyphal morphology was observed by a light microscope. Scale bars shown in the pictures are 10 µm. The hyphal tips of the two strains showed no differences from each other. (**D**) Agarose gel electrophoresis analysis of dsRNA extracted from the strains YC81-2 and P131. dsRNA samples were loaded into a 1.5% agarose gel. Multiple bands were observed in the dsRNA sample of YC81-2.

The morphological characteristics of the strains YC81-2 and P131 on PDA were observed. The colony of strain YC81-2 was black and flat, with little mycelia. The colony of strain P131 was grayish white and flat, with dense mycelia (Figure 1A). There was no significant difference in the mycelial growth rate and hyphal tip morphology (Figure 1B,C). Using agarose gel electrophoresis analysis, multiple bands were observed in the dsRNA sample of strain YC81-2, while no band was detected in the dsRNA sample of virus-free strain P131 (Figure 1D). This result indicates that strain YC81-2 might be infected with several RNA viruses.

### 3.2. Four Novel (+) ssRNA Magnaporthe Oryzae Botourmiaviruses

**Figure 2:**
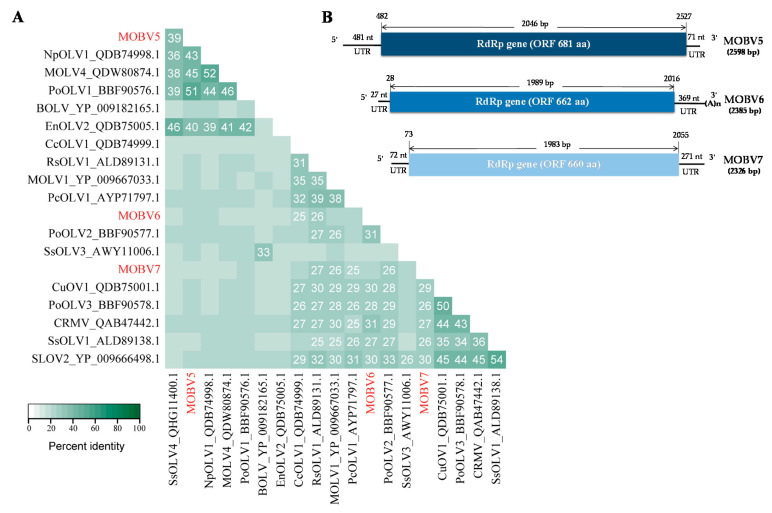
Analyses of three newly identified (+) ssRNA viral genomes and viruses belonging to the family *Botourmiaviridae* and the viral genome organization. (**A**) Percent identity matrix of the viral RdRP sequences generated by Clustal-Omega 2.1. Cutoff values were 25%. The viruses identified in this study are represented in red. (**B**) Schematic representation of the MOBV5, MOBV6, and MOBV7 genome organizations. Each virus contained a single open reading frame (ORF) encoding a putative RNA-dependent RNA polymerase (RdRp), and 5′and 3′ untranslated regions (UTRs).

To identify the bands in strain YC81-2, high-throughput sequencing was carried out. Via virome sequencing of YC81-2, a total of six mycoviruses were detected, including an identified virus in the family *Tombusviridae*, Magnaporthe oryzae virus A (MoVA), one novel virus in the family *Narnaviridae*, named as Magnaporthe oryzae narnavirus virus 2 (MoNV2), and four novel viruses in the family *Botourmiaviridae*. Based on the alignment with non-redundant protein sequence collection in the NCBI database using BLASTp, the RdRp of one novel botourmiavirus displayed the highest identity with the RdRp of MOLV1. Thus, this virus was classified in the genus *Magoulivirus* and designated as Magnaporthe oryzae botourmiavirus 2 (MOBV2). The other three novel botourmiaviruses were selected for further study. Since five botourmiaviruses (previously named ourmia-like mycoviruses), MOLV1, MOLV4, PoOLV1, PoOLV2, and PoOLV3, had been found in *M. oryzae*, the viruses found in this study were designated as MOBV5, MOBV6, and MOBV7, respectively. The 5′- and 3′-ends of each viral cDNA were determined using ligase-mediated terminal amplification (Figure 2B). The partial sequences of MoNV2 and MOBV2 were submitted to NCBI with the accession numbers provided as MW117114 and MW117115. Molecular characterization of the whole viral genomes is still underway.

#### 3.2.1. Molecular Characterization of the Three Botourmiaviruses

The complete nucleotide sequences of MOBV5, MOBV6, and MOBV7 were assembled by DNAMAN. The schematic representations of their genomic organization are shown in Figure 2B. The full-length cDNA sequences of MOBV5, MOBV6, and MOBV7 were deposited in the Genbank database under accession numbers MN648455, MN971591, and MN971592, respectively. The three mycoviruses were previously designated as Magnaporthe oryzae ourmia-like mycoviruses 5, 6, and 7 (MOLV5, MOLV6, and MOLV7) when we uploaded the information of the three mycoviruses to NCBI. In this study, ICTV MOLV5, MOLV6, and MOLV7 were modified to MOBV5, MOBV6, and MOBV7, respectively, according to ICTV recommendations.

#### 3.2.2. MOBV5

Sequence analysis of the full-length cDNA indicated that the MOBV5 genome (Figure 2B) was a (+) ssRNA molecule with a length of 2598 nts and a C+G content of 53.8%. ORF prediction revealed that MOBV5 consisted of a unique ORF of 2046 nts (nt positions 482–2527), which encoded a 681 amino acid (aa) residue polypeptide with a calculated molecular mass of 76.5 kDa. The 5′- untranslated region (UTR) of MOBV5 was determined to be 481 nts long. The 3′-UTR was relatively short, at only 71 nts long. BLASTp alignment of its RdRp indicated that MOBV5 showed the highest (E-value: 0; query cover: 75%) amino acid identity (51%) with Pyricularia oryzae ourmia-like virus 1 (PoOLV1) (Figure 2A).

#### 3.2.3. MOBV6

MOBV6 was also found to be a (+) ssRNA virus (Figure 2B). It was determined that the full-length nucleotide sequence of its cDNA was 2385 nts in length and the C+G content was 55.3%. Like MOBV5, MOBV6 was also composed of a 1989 nt unique putative ORF from nt positions 28 to 2016, that encoded a deduced protein of 662 aa residues with a calculated molecular mass of 72.73 kDa. Its 5′- UTR and 3′-UTR were 27 and 369 nts, respectively. The RdRp of MOBV6 showed the highest sequence similarity to that of Pyricularia oryzae ourmia-like virus 2 (PoOLV2, identity: 34%, E-value: 9 × 10^−16^; query cover: 30%), as shown in Figure 2A.

#### 3.2.4. MOBV7

The complete nucleotide sequence of MOBV7′s cDNA was 2326 nts in length (Figure 1E). The C+G content was 52.7%. There was only one ORF of 1983 nt (nt positions 73–2055) in the genome of MOBV7. The ORF encoded a polypeptide of 660 aa residues with a calculated molecular mass of 75.4 kDa. The 5′ and 3′-UTRs of MOBV7 were 72 and 271 nts in length, respectively. The RdRp of MOBV7 was most closely related to Soybean leaf-associated ourmiavirus 2 (SLOV2, identity: 30%, E-value: 4 × 10^−42^; query cover: 64%) in the genus *Scleroulivirus* (Figure 2A).

### 3.3. Predicted Secondary Structures of the 5′ and 3′ Terminal Regions

**Figure 3:**
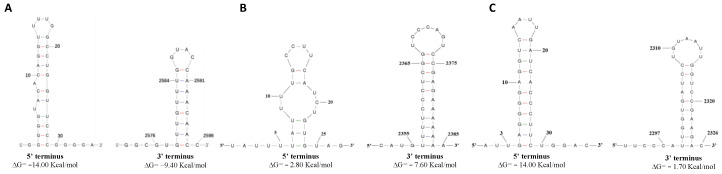
Predicted secondary structures of the MOBV5, MOBV6, and MOBV7 terminal sequences. (**A**) The predicted secondary structures of the 5′- and 3′- terminal sequences of MOBV5. (**B**) The predicted secondary structures of the 5′- and 3′- terminal sequences of MOBV6. (**C**) The predicted secondary structures of the 5′- and 3′- terminal sequences of MOBV7. The RNAfold server was used for predicting the secondary structures of the termini, and for calculating the free energies as well. The 5′ and 3′-terminal sequences of each virus are folded to form potential stable stem-loop structures.

The potential terminal stable stem-loop secondary structures of the 5′ and 3′-terminal sequences of MOBV5, MOBV6, and MOBV7 were predicted via the RNAfold web server. The 5′-terminal sequence (nt positions 1–30) and the 3′-terminal sequence (nt positions 2576–2598) of MOBV5 could be folded into terminal stable stem-loop structures with ΔG values of −14.00 and −9.40 kcal/mol, respectively (Figure 3A). The MOBV6 5′-terminal sequence (nt positions 5–25) and the 3′-terminal sequence (nt positions 2355–2385) could be folded into terminal stable stem-loop structures with ΔG values of −2.80 and −7.60 kcal/mol, respectively (Figure 3B). Both the 5′-terminus (nt positions 3–30) and the 3′-terminus (nt positions 2297–2326) of MOBV7 could be folded into potential stem-loop structures with a ΔG values of −14.00 and −1.70 kcal/mol, respectively (Figure 3C).

### 3.4. Alignment and Phylogenetic Analysis of Viral RdRps

**Figure 4:**
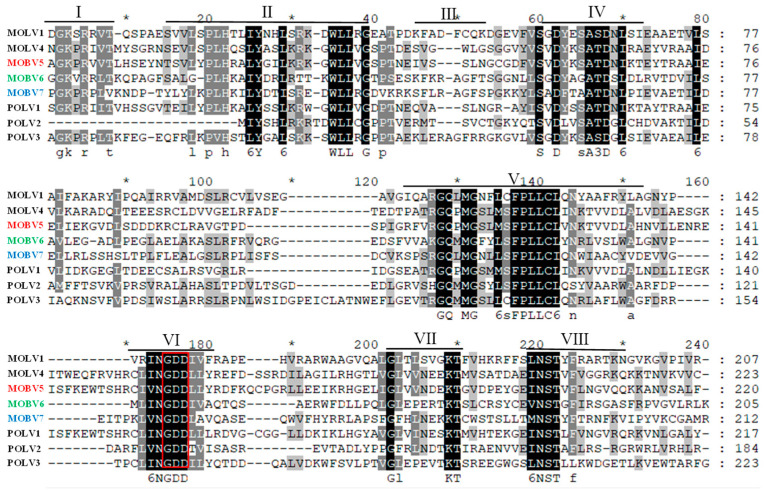
Multiple alignment of the amino acid sequences of RdRp encoded by MOBV5, MOBV6, and MOBV7 and previously identified Magnaporthe oryzae ourmia-like viruses (MOLV1 and MOLV4). The conserved motifs in the RdRp of the compared viruses are shown by roman numerals I to Ⅷ. Identical residues are color-highlighted with a black shadow, while conserved and semi-conserved amino acid residues are colored-highlighted with a gray shadow. The red box in domain VI showed the highly conserved GDD motifs in botourmiaviruses. The multiple sequence alignment was performed using the CLUSTALX 1.8 program.

Based on multiple alignments and a conserved domain database search with the previously identified Magnaporthe botourmiaviruses, typical conserved motifs of the RdRps of (+) ssRNA mycoviruses [51], including the highly conserved core domain GDD in motif VI, were identified in MOBV5, MOBV6 and MOBV7 (Figure 4).

**Figure 5:**
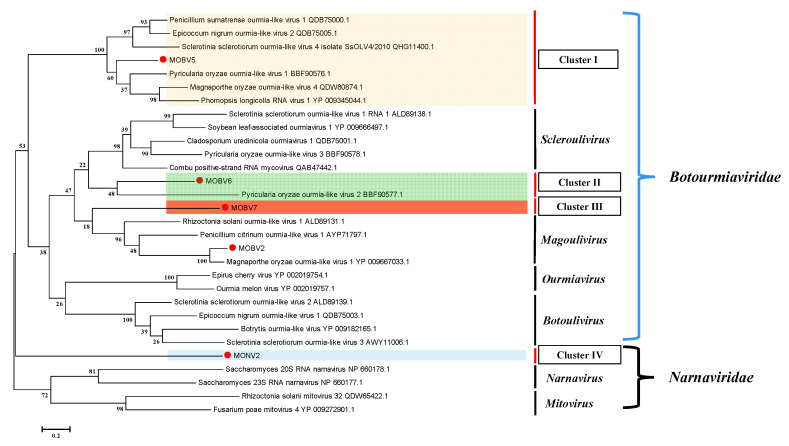
A maximum likelihood phylogenetic tree constructed based on an alignment of the respective RdRp amino acid sequences of MOBV2, MOBV5, MOBV6, MOBV7, and MoNV2 against other members of the families *Botourmiaviridae* and *Narnaviridae*. There were four established genera in the family *Botourmiaviridae*. MOBV2 was grouped into the genus *Magoulivirus*, while MOBV5, MOBV6, and MOBV7 (indicated with red dots) were grouped into three different clusters with certain unclassified Botourmiaviruses. Bootstrap values (%) obtained with 1000 replicates are indicated on branches. The names and GenBank accession numbers of other viruses are shown.

To define the evolutionary relationship of MOBV2, MOBV5, MOBV6, and MOBV7, a phylogenetic tree was constructed using the maximum likelihood (ML) method with 1000 bootstrap interactions for the complete RdRp amino acid sequences of viruses from the families *Botourmiaviridae* and *Narnaviridae* (Figure 5). The ML phylogenetic tree displayed three major clades, of which the first two clades grouped viruses in the family *Naranaviridae* (*mitovirus* and *narnavirus*), while the third clade embraced viruses of the new proposed and accepted family *Botourmiaviridae*. Botourmiaviruses were clustered into four classified genera and two unclassified groups in the phylogenetic tree. The phylogenetic tree showed that MOBV5, MOBV6, and MOBV7 indeed belonged to the family *Botourmiaviridae,* but were not clustered into any classified genera. They were relatively distant from MOLV1, which belongs to the genus *Magoulivirus*. MOBV2 was grouped into the genus Magoulivirus. The results suggest that new genera should be supposed in the family *Botourmiaviridae* to embrace the new members.

## 4. Discussion

In this study, four novel (+) ssRNA mycoviruses (MOBV2, MOBV5, MOBV6, and MOBV7) that co-infect an isolate of the plant pathogenic fungus *M. oryzae* were identified via virome sequencing. Of them, the RdRp of MOBV2 displayed 79% identity with that of MOLV1 and belongs to the genus *Magoulivirus*. Using ligase-mediated terminal amplification, the full-length nucleotide sequences of MOBV5, MOBV6, and MOBV7 cDNAs were determined as 2598, 2385, and 2326 nts, respectively. The genome of each virus consists of a single unique ORF. The proteins encoded by MOBV5, MOBV6, and MOBV7 show 52%, 34%, and 34% amino acid sequence identity with RdRps of three botourmiaviruses in the family *Botourmiaviridae*; PoOLV1, PoOLV2, and SLOV2, respectively. Multiple alignments with the RdRps of certain botourmiaviruses showed that these proteins contain conserved domains of the RdRp in (+) ssRNA viruses (motifs I-VIII) [22,51], including the highly conserved core domain GDD (motif VI). Moreover, phylogenetic analysis demonstrated that MOBV5, MOBV6, and MOBV7 belong to the family *Botourmiaviridae,* but are significantly distant from known *Botourmiaviruses*, and are not clustered into any classified genera in this family.

The family *Botourmiaviridae* was established by ICTV in 2018, and includes viruses that infect plants and filamentous fungi containing a (+) ssRNA genome that could be mono- or multi-segmented. It consists of four genera: *Ourmiavirus* (plant viruses), *Botoulivirus*, *Magoulivirus*, and *Scleroulivirus* (fungal viruses). Members of the genera *Botoulivirus*, *Magoulivirus*, and *Scleroulivirus* that infect fungi have a genome with a single segment of 2000–3200 nts encoding an RdRp. The demarcation criteria for these three mycovirus genera are based on differences in the RdRp amino acid sequence. Members of different genera in the family *Botourmiaviridae* should be >70% different in their complete RdRp amino acid sequence. However, in the last two years, several ourmia-like viruses that could not be classified into any of these three established genera have also been reported, such as SsOLV4, PoOLV1, and MOLV4. The identities of amino acid sequences of these are all lower than 30% compared with representative mycoviruses (BOLV, MOLV1 and SsOLV1) in three established genera [23,24], which indicates the underestimated viral diversity in the family *Botourmiaviridae*. Our results also demonstrate that compared with BOLV, MOLV1, or SsOLV1, the identities of the amino acid sequences of MOBV5, MOLV6, and MOLV7 were all close to or lower than 30%. We also compared the identities of amino acid sequences between MOBV5 and MOBV6 (28%), MOBV5 and MOBV7 (27%), and MOBV6 and MOBV7 (31%). Taken together, these results indicate that MOVB5, MOBV6, and MOBV7 cannot be classified into any of the three established genera, and each of them may represent a new genus.

Due to the simple single strand structure, the botourmiaviruses in the family *Botourmiaviridae* might evolve quickly and have diverse functions. Their terminal sequences exhibit several different structural features. For example, MOLV1 and MOLV4 have 3′- and 5′-UTRs with variable lengths and sequences, which can be folded into a stable stem-loop secondary structure. BOLV contains stretches of 28 and 50 nt sequences at the 5′- and 3′- non coding regions (NCRs) that can form a slightly less stable stem-loop structure [52]. Furthermore, the 5′- and 3′-terminuses of Sclerotinia sclerotiorum ourmia-like virus 4 (SsOLV4) harbor a G-pentamer (GGGGG) and a C-pentamer (CCCCC), respectively, which is similar to the narnaviruses in *Saccharomyces* [53]. The typical botourmiaviruses are not polyadenylated at their 3′-NCR. However, MOLV1, a representative isolate of the genus *Magoulivirus*, has a poly (A) tail at its 3′-end. In mycoviruses, this structure was first reported in SsMV2/KL-1, a mitovirus infecting *Sclerotinia sclerotiorum* [54], and was hypothesized to provide a recognition site for RdRp and/or protect the virus genome from fungal enzymatic degradation [55,56]. Moreover, the predicted secondary structure of PoOLV1 RNA indicated that its 5′-NCR might be folded into two stable stem loop structures, with a region matching with its associated RNA (ARNA), which would facilitate the recognition of other segments. The 3′-NCR of PoOLV3 also shares a stretch of 255 nt with ARNA3 at the 5′ half region [24]. Our present study supported the hypothesis that the 5′- and 3′-ends of botourmiaviruses are varied. Two NCRs of MOBV5, MOBV6, and MOBV7 can be folded into stable stem-loop secondary structures. The length of the 3′-UTRs of MOBV5, MOBV6, and MOBV7 are 71, 369, and 271 nts, respectively, which are longer than that of MOLV4 (29 nts), but shorter than that of MOLV1 (430 nts). Although MOBV5, with a 481 nt 5′-UTR, exhibited a high identity with PoOLV1, no ARNA was detected in our sequencing data. Furthermore, MOBV6 also has a seven nt poly (A) tail at its 3′-terminal. In addition to MOLV1, it is the second botourmiavirus that has been found to be polyadenylated at the 3′-NCR in the family *Botourmiaviridae* associated with *M. oryzae*.

So far, none of the mycoviruses in the family *Botourmiaviridae* have been found to be associated with fungal hypovirulence. However, mitoviruses that belong to the genus *Narnaviridae* and are closely related to botourmiavirus have been found to be relevant to the variable virulence of their fungal hosts. For example, Cryphonectria cubensis mitovirus 1b and Cryphonectria cubensis mitovirus 2a have no significant impact on their hosts [57]. On the other hand, Botrytis cinereal mitovirus 1 (BcMV1) can strongly debilitate its host and reduce its pathogenicity [58]. The co-infection of several mitoviruses also confers hypovirulence to their hosts [59]. Whether there are any hypovirulent or hypervirulent mycoviruses in the family *Botourmiaviridae* is still unknown. It has been reported that certain mycoviruses infecting *M. oryzae*, such as MoCV1-C, MoV3, and MOPV2, have been successfully removed from their fungal hosts through single-spore isolation. However, in the present study, we failed to generate any virus-free strains of YC81-2 using the same strategy. This result indicates that MOBV2, MOBV5, MOBV6, and MOBV7 might vertically transfect via spores. In follow-up research, we will try to obtain virus-free strains using other methods, such as protoplast regeneration technology, to determine the impact of the four botourmiaviruses investigated here on host pathogenicity and the interactions among themselves.

## Supplementary Materials

The following are available online at https://www.mdpi.com/1999-4915/12/12/1383/s1, Table S1: The primers used in the experiment.

## References

[1] Pearson M.N., Beever R.E., Boine B., Arthur K. (2009). Mycoviruses of filamentous fungi and their relevance to plant pathology. Mol. Plant Pathol..

[2] Ahn I.-P., Lee Y.-H. (2001). A viral double-stranded RNA up regulates the fungal virulence of *Nectria radicicola*. Mol. Plant-Microbe Interact..

[3] Jian J., Lakshman D.K., Tavantzis S.M. (1997). Association of distinct double-stranded RNAs with enhanced or diminished virulence in *Rhizoctonia solani* infecting potato. Mol. Plant-Microbe Interact..

[4] Ghabrial S.A., Suzuki N. (2009). Viruses of plant pathogenic fungi. Annu. Rev. Phytopathol..

[5] Ghabrial S.A., Castón J.R., Jiang D., Nibert M.L., Suzuki N. (2015). 50-plus years of fungal viruses. Virology.

[6] Donaire L., Pagán I., Ayllón M.A. (2016). Characterization of *Botrytis cinerea* negative-stranded RNA virus 1, a new mycovirus related to plant viruses, and a reconstruction of host pattern evolution in negative-sense ssRNA viruses. Virology.

[7] Yu X., Li B., Fu Y., Jiang D., Ghabrial S.A., Li G., Peng Y., Xie J., Cheng J., Huang J. (2010). A geminivirus-related DNA mycovirus that confers hypovirulence to a plant pathogenic fungus. Proc. Natl. Acad. Sci. USA.

[8] Krupovic M., Ghabrial S.A., Jiang D., Varsani A. (2016). *Genomoviridae*: A new family of widespread single-stranded DNA viruses. Arch. Virol..

[9] Yu X., Li B., Fu Y., Xie J., Cheng J., Ghabrial S.A., Li G., Yi X., Jiang D. (2013). Extracellular transmission of a DNA mycovirus and its use as a natural fungicide. Proc. Natl. Acad. Sci. USA.

[10] Liu S., Xie J., Cheng J., Li B., Chen T., Fu Y., Li G., Wang M., Jin H., Wan H. (2016). Fungal DNA virus infects a mycophagous insect and utilizes it as a transmission vector. Proc. Natl. Acad. Sci. USA.

[11] King A., Adams M., Carstens E., Lefkowitz E. (2012). Virus Taxonomy: Classification and Nomenclature of Viruses: Ninth Report of the International Committee on Taxonomy of Viruses.

[12] Yamashita S., Doi Y., Yora K. (1971). A polyhedral virus found in rice blast fungus, *Pyricularia oryzae* Cavara. Jpn. J. Phytopathol..

[13] Maejima K., Himeno M., Komatsu K., Kakizawa S., Yamaji Y., Hamamoto H., Namba S. (2008). Complete nucleotide sequence of a new double-stranded RNA virus from the rice blast fungus, *Magnaporthe oryzae*. Arch. Virol..

[14] Tang L., Hu Y., Liu L., Wu S., Xie J., Cheng J., Fu Y., Zhang G., Ma J., Wang Y. (2015). Genomic organization of a novel victorivirus from the rice blast fungus *Magnaporthe oryzae*. Arch. Virol..

[15] Yokoi T., Yamashita S., Hibi T. (2007). The nucleotide sequence and genome organization of Magnaporthe oryzae virus 1. Arch. Virol..

[16] Higashiura T., Katoh Y., Urayama S.-i., Hayashi O., Aihara M., Fukuhara T., Fuji S.-i., Kobayashi T., Hase S., Arie T. (2019). Magnaporthe oryzae chrysovirus 1 strain D confers growth inhibition to the host fungus and exhibits multiform viral structural proteins. Virology.

[17] Urayama S., Kato S., Suzuki Y., Aoki N., Le M.T., Arie T., Teraoka T., Fukuhara T., Moriyama H. (2010). Mycoviruses related to *chrysovirus* affect vegetative growth in the rice blast fungus *Magnaporthe oryzae*. J. Gen. Virol..

[18] Urayama S.-i., Sakoda H., Takai R., Katoh Y., Le T.M., Fukuhara T., Arie T., Teraoka T., Moriyama H. (2014). A dsRNA mycovirus, Magnaporthe oryzae chrysovirus 1-B, suppresses vegetative growth and development of the rice blast fungus. Virology.

[19] Du Y., He X., Zhou X., Fang S., Deng Q. (2016). Complete nucleotide sequence of Magnaporthe oryzae partitivirus 1. Arch. Virol..

[20] Chen W., Liang K., Li Y., Xie J., Chen J., Fu Y. (2017). Characterization of a novel partitivirus in the phytopathogenic fungus *Magnaporthe oryzae*. Acta Pharmacol. Sin..

[21] Ai Y.-P., Zhong J., Chen C.-Y., Zhu H.-J., Gao B.-D. (2016). A novel single-stranded RNA virus isolated from the rice-pathogenic fungus *Magnaporthe oryzae* with similarity to members of the family *Tombusviridae*. Arch. Virol..

[22] Illana A., Marconi M., Rodríguez-Romero J., Xu P., Dalmay T., Wilkinson M.D., Ayllón M.Á., Sesma A. (2017). Molecular characterization of a novel ssRNA ourmia-like virus from the rice blast fungus *Magnaporthe oryzae*. Arch. Virol..

[23] Li C.X., Zhu J.Z., Gao B.D., Zhu H.J., Zhou Q., Zhong J. (2019). Characterization of a novel ourmia-like mycovirus Infecting *Magnaporthe oryzae* and implications for viral diversity and evolution. Viruses.

[24] Ohkita S., Lee Y., Nguyen Q., Ikeda K., Suzuki N., Nakayashiki H. (2019). Three ourmia-like viruses and their associated RNAs in *Pyricularia oryzae*. Virology.

[25] Lin Y., Zhou J., Zhou X., Shuai S., Zhou R., An H., Fang S., Zhang S., Deng Q. (2020). A novel narnavirus from the plant-pathogenic fungus *Magnaporthe oryzae*. Arch. Virol..

[26] Wu S., Cheng J., Fu Y., Chen T., Jiang D., Ghabrial S.A., Xie J. (2017). Virus-mediated suppression of host non-self recognition facilitates horizontal transmission of heterologous viruses. PLoS Pathog..

[27] Chiba S., Suzuki N. (2015). Highly activated RNA silencing via strong induction of dicer by one virus can interfere with the replication of an unrelated virus. Proc. Natl. Acad. Sci. USA.

[28] Ikeda K., Inoue K., Kida C., Uwamori T., Sasaki A., Kanematsu S., Park P. (2013). Potentiation of mycovirus transmission by zinc compounds via attenuation of heterogenic incompatibility in *Rosellinia necatrix*. Appl. Environ. Microbiol..

[29] Thapa V., Roossinck M.J. (2019). Determinants of coinfection in the mycoviruses. Front. Cell Infect. Microbiol..

[30] Hillman B.I., Annisa A., Suzuki N., Kielian M., Mettenleiter T.C., Roossinck M.J. (2018). Chapter five—Viruses of plant-interacting fungi. Advances in Virus Research.

[31] Sun L., Nuss D.L., Suzuki N. (2006). Synergism between a mycoreovirus and a hypovirus mediated by the papain-like protease p29 of the prototypic hypovirus CHV1-EP713. J. Gen. Virol..

[32] Sasaki A., Nakamura H., Suzuki N., Kanematsu S. (2016). Characterization of a new megabirnavirus that confers hypovirulence with the aid of a co-infecting partitivirus to the host fungus, *Rosellinia necatrix*. Virus Res..

[33] Zhang D.-X., Nuss D.L. (2016). Engineering super mycovirus donor strains of chestnut blight fungus by systematic disruption of multilocus *vic* genes. Proc. Natl. Acad. Sci. USA.

[34] Nerva L., Ciuffo M., Vallino M., Margaria P., Varese G.C., Gnavi G., Turina M. (2016). Multiple approaches for the detection and characterization of viral and plasmid symbionts from a collection of marine fungi. Virus Res..

[35] Osaki H., Sasaki A., Nomiyama K., Tomioka K. (2016). Multiple virus infection in a single strain of *Fusarium poae* shown by deep sequencing. Virus Genes.

[36] Kozlakidis Z., Herrero N., Ozkan S., Bhatti M.F., Coutts R.H.A. (2013). A novel dsRNA element isolated from the *Aspergillus foetidus* mycovirus complex. Arch. Virol..

[37] Sun L., Suzuki N. (2008). Intragenic rearrangements of a mycoreovirus induced by the multifunctional protein p29 encoded by the prototypic hypovirus CHV1-EP713. RNA.

[38] Tanaka T., Sun L., Tsutani K., Suzuki N. (2011). Rearrangements of mycoreovirus 1 S1, S2 and S3 induced by the multifunctional protein p29 encoded by the prototypic hypovirus Cryphonectria hypovirus 1 strain EP713. J. Gen. Virol..

[39] Eusebio-Cope A., Suzuki N. (2015). Mycoreovirus genome rearrangements associated with RNA silencing deficiency. Nucleic Acids Res..

[40] Kashif M., Jurvansuu J., Vainio E.J., Hantula J. (2019). Alphapartitiviruses of *Heterobasidion* wood decay fungi affect each other’s transmission and host growth. Front. Cell Infect. Microbiol..

[41] Vainio E.J., Müller M.M., Korhonen K., Piri T., Hantula J. (2015). Viruses accumulate in aging infection centers of a fungal forest pathogen. ISME J..

[42] Xue M., Yang J., Li Z., Hu S., Yao N., Dean R.A., Zhao W., Shen M., Zhang H., Li C. (2012). Comparative analysis of the genomes of two field isolates of the rice blast fungus *Magnaporthe oryzae*. PLoS Genet..

[43] Morris T.J., Dodds J.A. (1979). Isolation and analysis of double-stranded RNA from virus-infected plant and fungal tissue. Phytopathology.

[44] Potgieter A., Page N., Liebenberg J., Wright I., Landt O., Van Dijk A. (2009). Improved strategies for sequence-independent amplification and sequencing of viral double-stranded RNA genomes. J. Gen. Virol..

[45] Thompson J.D., Gibson T.J., Plewniak F., Jeanmougin F., Higgins D.G. (1997). The CLUSTAL_X windows interface: Flexible strategies for multiple sequence alignment aided by quality analysis tools. Nucleic Acids Res..

[46] Nicholas K.B., Nicholas H.B., Deerfield D.W. (1997). GeneDoc: Analysis and visualization of genetic variation. Embnew. News.

[47] Gilbert K.B., Holcomb E.E., Allscheid R.L., Carrington J.C. (2019). Hiding in plain sight: New virus genomes discovered via a systematic analysis of fungal public transcriptomes. PLoS ONE.

[48] Li W., Cowley A., Uludag M., Gur T., McWilliam H., Squizzato S., Park Y.M., Buso N., Lopez R. (2015). The EMBL-EBI bioinformatics web and programmatic tools framework. Nucleic Acids Res.

[49] Tamura K., Stecher G., Peterson D., Filipski A., Kumar S. (2013). MEGA6: Molecular evolutionary genetics analysis version 6.0. Mol. Biol. Evol..

[50] RNAfold WebServer. http://rna.tbi.univie.ac.at/cgi-bin/RNAWebSuite/RNAfold.cgi.

[51] Koonin E.V. (1991). The phylogeny of RNA-dependent RNA polymerases of positive-strand RNA viruses. J. Gen. Virol..

[52] Donaire L., Rozas J., Ayllón M.A. (2016). Molecular characterization of Botrytis ourmia-like virus, a mycovirus close to the plant pathogenic genus *Ourmiavirus*. Virology.

[53] Wang Q., Mu F., Xie J., Cheng J., Fu Y., Jiang D. (2020). A single ssRNA segment encoding RdRp is sufficient for replication, infection, and transmission of Ourmia-Like virus in fungi. Front. Microbiol..

[54] Xie J., Ghabrial S.A. (2012). Molecular characterizations of two mitoviruses co-infecting a hyovirulent isolate of the plant pathogenic fungus *Sclerotinia sclerotiorum*. Virology.

[55] Hong Y., Cole T.E., Brasier C.M., Buck K.W. (1998). Evolutionary relationships among putative RNA-dependent RNA polymerases encoded by a mitochondrial virus-like RNA in the dutch elm disease fungus, Ophiostoma novo-ulmi, by other viruses and virus-like RNAs and by the *Arabidopsis Mitochondrial* genome. Virology.

[56] Hong Y., Dover S.L., Cole T.E., Brasier C.M., Buck K.W. (1999). Multiple mitochondrial viruses in an isolate of the *Dutch Elm* disease fungus Ophiostoma Novo-Ulmi. Virology.

[57] Van Heerden S.W. (2008). Studies on Cryphonectria Cubensis in South Africa with Special Reference to Mycovirus Infection.

[58] Wu M., Zhang L., Li G., Jiang D., Hou M., Huang H.-C. (2007). Hypovirulence and double-stranded RNA in *Botrytis cinerea*. Phytopathology.

[59] Khalifa M.E., Pearson M.N. (2013). Molecular characterization of three mitoviruses co-infecting a hypovirulent isolate of *Sclerotinia sclerotiorum* fungus. Virology.

