# Supplementary material for: Four Novel Botourmiaviruses Co-Infecting an Isolate of the Rice Blast Fungus Magnaporthe oryzae
Source: Viruses. 2020 Dec 3;12(12):1383. doi: 10.3390/v12121383 (PMC7761653; doi:10.3390/v12121383)
Supplement: Supplementary file 1 [file viruses-12-01383-s001.pdf]

Supplementary TableS1 The primers used in the experiment

Primer name	Sequences (5' to 3')
MOBV5-F1	GCGAGGAAGAGTGTATCGCT
MOBV5-F2	TGCTCGGGAAACAGGATTGT
MOBV5-R1	TAAGGCGATGGCTCTGTGAG
MOBV5-R2	CCTGAACGACATTTCTGCCG
MOBV6-F1	ACTTCCCTCCTCGCAAAATC
MOBV6-F2	CCTCCTCGCAAAATCCAGAA
MOBV6-R1	GGTCAACGAGTGGGATTACC
MOBV6-R2	GCTTAGTCTTCCGCCTTCTC
MOBV7-F1	GTGTCGACGACGGAGATGAT
MOBV7-F2	TGTGAGCTGTGTTCTCGTGT
MOBV7-R1	ACGTAACCTCCTCTGGGTCT
MOBV7-R2	CTTTGGAACCGAAGCCAGTG
PC2	CCGAATTCCCGGGATCC
